# The relationship between workplace violence, anxiety, and depression in nurses during the COVID-19 pandemic

**DOI:** 10.47626/1679-4435-2024-1337

**Published:** 2025-08-25

**Authors:** Ana Paula Nogueira de Magalhães, Diego de Oliveira Souza, Sónia Mafalda Pereira Ribeiro

**Affiliations:** 1 Complexo de Ciências Médicas e Enfermagem, Universidade Federal de Alagoas, Campus Arapiraca, Arapiraca, AL, Brazil; 2 Instituto Superior Miguel Torga, Centro Lusíada de Investigação em Serviço Social e Intervenção Social, Coimbra, Portugal.

**Keywords:** workplace violence, nursing, team, anxiety, depression, occupational health., violência no trabalho, equipe de enfermagem, ansiedade, depressão, saúde ocupacional.

## Abstract

**Introduction:**

Workplace violence severely affects nurses, and the COVID-19 pandemic has intensified this issue, resulting in physical and mental illness, which seriously affects their work and lives.

**Objectives:**

To analyze the relationship between workplace violence, anxiety, and depression among front line nurses in the fight against COVID-19.

**Methods:**

This mixed-methods study simultaneously collected qualitative and quantitative data in three municipalities in Alagoas, Brazil, between January and June 2022. The qualitative data were derived from interviews with six nurses, using the thematic oral history technique. The quantitative data were derived from a questionnaire administered to 131 clinical and practical nurses.

**Results:**

The oral histories indicated anxiety-related feelings, such as fear, nervousness, worry, and panic, which made the workers vulnerable to violence. According to the quantitative data, 53.4% (n = 70) of nurses experienced violence, primarily psychological (n = 69; 52.7%). Anxiety and depression symptoms were present in 21.4% (n = 28) and 30.5% (n = 40) of the nurses, respectively, and violence was significantly associated with both anxiety (p = 0.003) and depression (p = 0.004).

**Conclusions:**

The results show that nurses suffer a high prevalence of violence. The association between violence, anxiety, and depression reinforces the need for structural initiatives to combat violence and protect the lives of those who care for the health of the population.

## Abstract (pt)

**Introdução:**

A violência relacionada ao trabalho afeta gravemente os trabalhadores da enfermagem. A pandemia de covid-19 intensificou essa problemática, levando esses trabalhadores ao adoecimento físico e mental, com sérias implicações para seus trabalhos e suas vidas.

**Objetivos:**

Analisar a relação entre violência no trabalho, ansiedade e depressão entre trabalhadores da enfermagem da linha de frente contra a covid-19.

**Métodos:**

Estudo de métodos mistos, com coleta simultânea de dados qualitativos e quantitativos em três municípios de Alagoas, Brasil, entre janeiro e junho de 2022. Para os dados qualitativos, foram realizadas entrevistas com seis enfermeiras, utilizando a técnica da história oral temática. Para os dados quantitativos, aplicou-se um questionário com 131 enfermeiros e técnicos de enfermagem.

**Resultados:**

Os depoimentos das histórias orais evidenciam a presença de sentimentos relacionados à ansiedade, como medo, nervosismo, preocupação e pânico, o que deixou os trabalhadores vulneráveis à violência. Os dados quantitativos demonstram que 53,4% (n = 70) dos trabalhadores da enfermagem sofreram violência, sobretudo psicológica (n = 69; 52,7%). A ansiedade foi identificada em 21,4% (n = 28) dos trabalhadores da enfermagem, enquanto 30,5% (n = 40) apresentaram depressão. A violência apresentou associação significativa com a ansiedade (p = 0,003) e com a depressão (p = 0,004).

**Conclusões:**

Os resultados constatam a alta prevalência da violência sofrida pelos trabalhadores da enfermagem. As associações encontradas entre violência, ansiedade e depressão reforçam a necessidade da promoção de ações estruturais para o enfrentamento da violência e para a proteção da vida daqueles que cuidam da saúde da população.

## INTRODUCTION

Workplace violence has become more frequent, and its relevance for worker health is increasing. Workplace violence has been analyzed in relation to: (1) violent acts committed by individuals involved in the work process and (2) the inherently violent nature of degrading work, due to organizational issues, work conditions, and work dynamics.^1^ This study will focus on the former, i.e., violence against nurses committed by any individual directly or indirectly involved in the work process. Although structural violence appears in an underlying form, the two dimensions are intertwined.

The health sector is notorious for workplace violence, especially among nurses,^2^ the health profession with the largest number of workers. Bordignon et al.^3^ report that nurses who are victims of violence are susceptible to a series of consequences in both their professional and personal lives.

Some of these consequences are related to mental health, such as stress, burnout, anxiety, and depression. In a Saudi Arabian study of 118 nurses, 56% reported having suffered at least one type of workplace violence, resulting in high levels of stress, emotional exhaustion, low personal fulfillment, and depersonalization, which suggests the presence of burnout.^4^

Pang et al.^5^ studied a sample of 6,771 Korean nurses, finding that verbal violence and sexual harassment increased depressive symptoms, which in turn increased turnover.^5^ In another study of 238 psychiatric nurses in Taiwan who had experienced violence, 75.9% had depressive symptoms.^6^

The uncertainty, stress, and precarious working conditions experienced during the COVID-19 pandemic (2020 to 2022) aggravated the problem of workplace violence, resulting in more cases of psychological violence, discrimination, and physical aggression among health care workers.^7,8^ Mental health problems, including anxiety and depression, also increased during this period through a multifaceted process that included greater workplace violence.^9^ A study of 532 nurses in southeastern Brazil corroborated this finding, intimidation and/or violence at work during the pandemic were associated with psychosis, obsessiveness/ compulsiveness, somatization, and anxiety.^10^

According to the above-cited studies, violence plays an important role in the illness process, especially in relation to mental health. Therefore, research that directly studies workplace violence, not only as a variable, but as an object of study, is relevant. In view of this, the present study analyzed the relationship between workplace violence, anxiety, and depression, among front line nurses during the COVID-19 pandemic.

## METHODS

This parallel-convergent mixed methods study compared quantitative and qualitative data to identify convergences, in a process of general interpretation of the results.^11^

This article is derived from “Risks and demands of/ at nursing work during the COVID-19 pandemic in Alagoas,” a study that included clinical and practical nurses who cared for COVID-19 patients for at least 1 month in hospitals in three cities in the state of Alagoas, Brazil, from April 2020 to April 2021. The 1-month inclusion criterion was due to the high turnover of workers during the pandemic, given that many had precarious employment relationships. Workers who were on vacation or work leave due to belonging to a risk group were excluded.

Only nurses were included in the qualitative stage. Six semi-structured interviews were conducted online using the Zoom and Google Meet platforms. The sample size was determined through information saturation. The interview script was prepared by the authors based on the thematic oral history method to contextualize the experience of working on the front lines against COVID-19, including workplace violence. Two undergraduate nursing students conducted the interviews after receiving training, guidance, and supervision from a nursing professor with experience in occupational health and qualitative research. The interviews were recorded, transcribed, and analyzed from the perspective of occupational health and historicaldialectical materialism, seeking to capture relevant details that converged with the quantitative data.

In the quantitative stage, cross-sectional data was collected from a convenience sample. Of the 681 workers invited to participate, 131 responded to the online questionnaire (19.2% response rate).^9,12^ The sample power was 87% (calculated *a posteriori*) based on the following parameters: n = 131, α = 0.05, and effect size = 0.3.

This study used the *Questionário Individual para Avaliação da Saúde dos Trabalhadores* (Individual Questionnaire for Worker Health Assessment), an instrument developed by the Worker Health Assessment and Monitoring Program (*Programa de Evaluación y Seguimiento de la Salud de los Trabajadores* - PROESSAT)^13^ after translation, adaption, and validation for speakers of Brazilian Portuguese.^14^ This instrument includes questions about living and working conditions and their health impact in the form of presumptive diagnoses.^13^

The research instrument and the informed consent form were uploaded as Google Forms and were sent to workers by email and/or WhatsApp between January 1 and June 30, 2022. The estimated response time was 3 weeks.

The dependent variable was “having suffered workplace violence during the pandemic” (yes, no). The following were considered independent variables: sociodemographic data (sex, age group, race), professional category (clinical nurse, practical nurse), violence type (verbal, psychological, physical, sexual), aggressor type (patients and/or family members, coworkers, superiors), in addition to presumptive diagnosis of anxiety (yes, no) and depression (yes, no).^13^ Presumptive diagnosis of anxiety disorder and depression was based on the feelings the workers reported on the questionnaire. The anxiety-related questions were: “Do you consider yourself easily irritable?”; “Do you feel afraid for no reason?”; “Do you have difficulty sleeping or staying asleep?”; “Do you worry excessively about insignificant setbacks?”; “Do you often feel worried?”; “Have you ever had any a problem with anxiety?”; and “Have you ever been treated at a mental hospital?” The depression-related questions were: “Do you often feel sad or unhappy?”; “Do you often want to die?”; “Are you very afraid of losing your job?”; “Does the future seem uncertain or hopeless to you?”; “Are you indifferent to violence?”; “Do you have a hard time starting a conversation in meetings?”; and “Do you prefer to ignore your problems?” According to PROESSAT recommendations,^13^ at least four positive answers were required in each section for a presumptive diagnosis of anxiety or depression.

Quantitative data analysis began by determining the frequency distribution of individual variables. Pearson’s chi-square test was used to identify associations between workplace violence, anxiety, and depression; the significance level was set at 5%. The data were analyzed in SPSS 25.0.

To combine the results, it was decided to begin with the qualitative data, because after analysis they were less specific regarding workplace violence. A summary of each oral history is presented, highlighting elements related to mental health and narratives of violence, allowing connections with the quantitative data.

Each participant in the qualitative stage was identified by the letter N and a number (i.e., N1 to N6) to ensure anonymity. This study conformed to National Health Council Resolutions 466/2012 and 510/2016 and was approved by the Universidade Federal de Alagoas Research Ethics Committee (decision 4,525,156). All workers provided written informed consent and participated voluntarily.

## RESULTS

The experience of each interviewed nurse is summarized below:

**N1:** After graduating in 2018, she was balancing her master’s studies with working on the front lines against COVID-19. She chose to work during the pandemic to make a contribution and gain experience. She reported difficulties with work overload, constant deaths, a lack of experience with the new disease, and isolation from her family.
**N2:** With 9 years of experience as a nurse, she considered the pandemic to be the greatest challenge of her career. She reported that the beginning of the pandemic was frightening, especially because they were facing an unknown enemy. Dealing with so many deaths, the isolation of her daughters, and managing the nursing team were extremely difficult. According to her, the intense psychological burden led to exhaustion among the nurses.
**N3:** After graduating 2 years ago, she immediately got a job at the hospital. She reported feeling that the profession is undervalued, although she enjoys the relationship with patients. She described the pandemic using words such as fear, ignorance, frightening, and traumatic. Especially at the beginning, she had to work double shifts due to the large number of patients and the shortage of nurses. Many professionals became sick, overloading staff who remained at work. She felt physically exhausted. Emotionally, she felt bad at the beginning, but felt better later on.
**N4:** Two months after graduation, she started working on the front lines against COVID-19, where she remained for 1 year and 7 months. She reported that when the first patients arrived, she was very scared, nervous, and panicked. It was a terrifying time because she didn’t know how to take care of people. It was very exhausting, with many tasks and insufficient resources. She believed that the worst part was controlling her emotions and dealing with the death of patients she knew.
**N5:** A nurse for 18 years, management asked her to work in the COVID-19 sector. She considered the beginning of the pandemic a terrible experience due to the lack of beds and nurses and large number of patients. She reported feeling scared and tired, since she only got 1 hour of rest during 24-hour shifts. She lived in fear of infecting her partner at home. She spent a lot of time away from her family and faced distressing days; she was reluctant to go to work.
**N6:** Having graduated 6 years ago, she reported liking her profession. She first took a technical course and then completed an undergraduate degree. She was working in a general hospital and worked on the front line against COVID-19 for 2 years. She reported that the worst period was prior to the beginning of vaccination, when there were many deaths, including young people. She felt tired and constantly worried about her family.

Thus, some of the statements indicate psychological suffering, such as characterizing the pandemic as frightening or terrible, in addition to feelings of fear, anguish, fatigue, exhaustion, psychological burden, worry, nervousness, and panic. Such a scenario would be conducive to violence, which, in turn, can heighten psychological suffering. One report describes a specific case of violence:

[...] *but the time came for the family members to arrive at the ICU and, without authorization, they invaded the ICU and said we killed their family member, that we were the ones who killed him, because yesterday he was fine and today he was dead. Even though we said that the patient was unstable and that his COVID progressed quickly [...] the families couldn’t understand sometimes. Families would come bursting in and say that we killed their relative, curse us, and say that they wished the ICU ceiling would fall on us. So, imagine us in this agony, this frustration, this work overload, this rush, and still having to deal with such scenes. It just wiped us out mentally.* (N4)

It should be acknowledged that the general population also faced a significant psychological burden during the pandemic, especially due to the fear of death and the loss of family members. N4’s report highlights this, showing how verbal and psychological violence becomes an additional psychological burden for nurses. To analyze the relationship between violence and psychological distress, quantitative data on violence, anxiety, and depression are presented below.

Of the 131 participants in the quantitative stage, 80.2% (n = 105) were women, 41.2% (n = 54) were between 30 and 39 years old, and 65.6% self-identified as mixed race (n = 86). Regarding professional category, 39.7% (n = 52) were clinical nurses and 60.3% (n = 79) were practical nurses (Table 1).

**Table 1:** Sociodemographic characteristics and violence suffered by nurses, Alagoas, Brazil, 2022 (n = 131)

Characteristics	n	%
Gender Female	105	80.2
Male	26	19.8
Other	-	-
Age range (years) < 20	-	-
20-29	31	23.7
30-39	54	41.2
40-49	40	30.5
50-59	5	3.8
≥ 60	1	0.8
Race Mixed	86	65.6
Black	14	10.7
White	27	20.6
Indigenous	-	-
Asian	4	3.1
Professional category Clinical Nurse	52	39.7
Practical Nurse	79	60.3
Suffered violence during the pandemic Yes	70	53.4
No	61	46.6
Suffered psychological violence Yes	69	52.7
No	62	47.3
Suffered verbal abuse Yes	53	40.5
No	78	59.5
Suffered physical violence Yes	5	3.8
No	126	96.2
Suffered sexual violence Yes	3	2.3
No	128	97.7
Suffered violence from patients and/or their family members Yes	48	36.6
No	83	63.4
Suffered violence from coworkers Yes	33	25.2
No	98	74.8
Suffered violence from superiors Yes	29	22.1
No	102	77.9
Suffered violence while commuting to work Yes	37	28.2
No	94	71.8

As shown in Table 1, 53.4% ( n = 70) of the nurses suffered some type of violence during the COVID-19 pandemic, especially psychological violence (52.7%). The main perpetrators were patients/family members (n = 48; 36.6%), followed by coworkers (n = 33; 25.2%) and superiors (n = 29; 22.1%).

Regarding psychological distress, 21.4% (n = 28) of the nurses had symptoms of anxiety, while 30.5% (n = 40) had symptoms of depression. According to the inferential analysis, workers who suffered violence had the highest prevalence of anxiety and depression; there was a positive association between workplace violence and anxiety (p = 0.003) and violence and depression (p = 0.004) (Table 2).

**Table 2:** The association between workplace violence, anxiety, and depression, Alagoas, Brazil, 2022 (n = 131)

Variables	Suffered workplace violence		p-value^*^
	Yes n (%)	No n (%)	
Anxiety			0.003
Yes	22 (78.6)	6 (21.4)	
No	48 (46.6)	55 (53.4)	
Depression			0.004
Yes	29 (72.5)	11 (27.5)	
No	41 (45.1)	50 (54.9)	

* Pearson’s chi-square test.

## DISCUSSION

The results of this study demonstrate that nurses suffered a high prevalence of violence during this period, and that there was an association between workplace violence, anxiety, and depression.

The precarious working conditions during the pandemic, associated with high patient mortality and social isolation resulted in intense physical and mental exhaustion for nurses. “This context led to psychological suffering and an increased possibility of exposure to workplace violence.”^15^ On the other hand, the violence may have triggered symptoms of anxiety and depression, creating a vicious cycle.

According to Seligmann-Silva,^15^ mental disorders resulting from psychological distress develop through work-related mental exhaustion and are, therefore, the result of workplace violence itself. Workplace violence is intertwined with structural violence, which is expressed through precarious working conditions, especially overload, long working hours, instability, and devaluation. These conditions have been associated with workplace violence, as well as different forms of psychological distress.^8,15^

In a cross-sectional study of 2,796 Chinese nurses,^16^ 49.12% (n = 1,360) reported suffering at least one episode of violence in the last 6 months. The following effects stood out: 1) decreased enthusiasm at work (n = 122; 65.24%); 2) anxiety, depression, and anger (n = 110; 58.82%); 3) intention to change careers (n = 71; 37.97%); 4) insomnia (n = 38; 20.32%); and 5) suicidal behavior (n = 6; 3.21%).

As noted, nurses were mainly affected by psychological violence. This type of violence includes threats, verbal abuse, harassment, and bullying^17^ and often appears in moments of acute anxiety, resulting from situations of extreme fatigue, when everyone is “on edge”,^15^ as in the context of the COVID-19 pandemic.

The oral histories of our sample describe anxietyrelated feelings, such as fear, irritation, worry, and panic. The tension and lack of emotional control experienced during the pandemic may have led to both violence and increased anxiety. A study of 1,030 Chinese health workers^18^ found that workplace violence had a significant impact on worker anxiety.

Regarding depression, exposure to violence can lead to mental fatigue and feelings such as frustration, loss of meaning, and self-devaluation, resulting in discouragement, sadness, slowed thinking, and difficulty participating in social activities.^15^

In the present study, nurses who experienced violence had a higher frequency of depression than those who did not. These findings corroborate previous findings of a strong relationship between violence and depressive symptoms.^19,20^ A prospective study of Danish workers^21^ found an association between workplace violence and depression 2 years after workers had suffered violence, demonstrating its longlasting consequences. These consequences include complex forms of suffering, such as burnout and behavioral problems, whose repercussions sometimes make it impossible to continue working.^6,10^

The main aggressors were patients or their family members. A Chinese study^22^ found that the main reasons for family member violence against health workers were patient deaths and dissatisfaction with treatment. Hospitalization is an intense process for patients and family members that leads to high levels of anxiety, stress, and suffering; the risk of violence increases if treatment expectations are not met.^23^ However, nurses also suffer violence from patients/ family members due to structural problems in the health system, such as precarious working conditions and lack of adequate resources.^8,24^

Work-related violence is exacerbated among nurses because the profession is predominantly female, i.e., the violence they face is intensified by inequality of power between the sexes, which is part of society in general and health care in particular. Throughout history, women have suffered violence in social spaces, and the same applies to nurses, since their workplace is marked by gender-based inequality and hierarchization.^23^

Some strategies have been developed to prevent violence against nurses. A study by the European Federation of Nurses Associations^8^ mapped actions, policies, and programs to combat violence. In Portugal, a national program has been in force since 2019 to raise awareness about the early detection of risks and antecedents of violence. In Finland and Switzerland, the phenomenon of violence against nurses has been included in the national nursing curriculum. In France, a closer link between hospitals and the police has been developed to increase safety: a contact person is designated for each hospital, and staff who suffer violence are supported after filing a complaint. Emergency departments are also actively monitored and the police can perform safety assessments for any health facility. The United Kingdom’s National Health System works with the police and the Crown Prosecution Service to help victims obtain evidence and provide quicker, more efficient legal proceedings. Staff are trained on how to deal with violence, and immediate mental health support is provided for staff who have been victimized.

In Brazil, strategies for confronting violence include training nurses to recognize situations of violence and promoting effective communication, in addition to creating institutional protocols that involve reception, listening spaces, victim referral, case notification, and protecting workers through specific laws.^10,25-27^ However, the persistent increase in workplace violence within the health system reinforces the need to for persistent collective strategies to address this phenomenon.^3^

This study has some limitations. First, the data were collected online during social isolation in the midst of the COVID-19 pandemic, which makes it difficult to generalize the results. Second, the cross-sectional nature of the quantitative data prevents determination of a causal relationship between workplace violence and anxiety and depression symptoms. However, the findings are consistent with the literature and are important for reflecting on the relationship between violence, mental suffering, and coping mechanisms.

## CONCLUSIONS

The results showed that nurses suffer a high prevalence of violence. The association between violence, anxiety, and depression indicates the need for initiatives to confront violence and protect the health and lives of caregivers. These initiatives should lead to strategies that address behavioral issues and, above all, organizational and structural issues, modifying work processes that create the conditions in which violence can occur.
